# Essential Oils in Cervical Cancer: Narrative Review on Current Insights and Future Prospects

**DOI:** 10.3390/antiox12122109

**Published:** 2023-12-13

**Authors:** Norhashima Abd Rashid, Nor Haliza Mohamad Najib, Nahdia Afiifah Abdul Jalil, Seong Lin Teoh

**Affiliations:** 1 Department of Biomedical Science, Faculty of Applied Science, Lincoln University College, Petaling Jaya 47301, Malaysia; norhashima@lincoln.edu.my; 2 Unit of Anatomy, Faculty Medicine & Health Defence, Universiti Pertahanan Nasional Malaysia, Kuala Lumpur 57000, Malaysia; norhaliza@upnm.edu.my; 3 Department of Anatomy, Faculty of Medicine, Universiti Kebangsaan Malaysia, Kuala Lumpur 56000, Malaysia; nahdia.afiifah@ukm.edu.my

**Keywords:** essential oil, natural product, phytomedicine, cancer, antiproliferative, drug delivery, nanoparticle

## Abstract

Cervical cancer is a prevalent and often devastating disease affecting women worldwide. Traditional treatment modalities such as surgery, chemotherapy, and radiation therapy have significantly improved survival rates, but they are often accompanied by side effects and challenges that can impact a patient’s quality of life. In recent years, the integration of essential oils into the management of cervical cancer has gained attention. This review provides an in-depth exploration of the role of various essential oils in cervical cancer, offering insights into their potential benefits and the existing body of research. The review also delves into future directions and challenges in this emerging field, emphasizing promising research areas and advanced delivery systems. The encapsulation of essential oils with solid lipid nanoparticles, nanoemulsification of essential oils, or the combination of essential oils with conventional treatments showed promising results by increasing the anticancer properties of essential oils. As the use of essential oils in cervical cancer treatment or management evolves, this review aims to provide a comprehensive perspective, balancing the potential of these natural remedies with the challenges and considerations that need to be addressed.

## 1. Introduction

Cervical cancer is the most common gynecologic malignancy and is the fourth most frequent cancer type among women globally, making it one of the main causes of mortality [1]. In 2020, it was estimated that 604,000 new cases and 342,000 deaths occurred among women due to this cancer [2]. Cervical cancer death rates differ from nation to nation, and about 90% of these occur in low- and middle-income countries due to limited access to preventive measures [2]. In addition, cervical cancer is always asymptomatic until it progresses to a severe phase. According to the World Health Organization [3], there is a lack of access to treatments for malignant lesions, such as chemotherapy, radiation, and surgery, which leads to an increase in the mortality rate from cervical cancer in these countries. The most common signs of cervical cancer include severe pelvic discomfort, extreme weight loss, and anorexia, as well as any abnormal discharge from the vagina (increased menstrual bleeding, bleeding between the two menstrual cycles, and post-menopausal bleeding) [4].

Surgery (e.g., radical hysterectomy and pelvic lymphadenectomy), radiation, and chemotherapy are the most often employed treatments for cervical cancer [5]. All these treatments, including chemotherapy, nevertheless, have exhibited serious adverse effects like bleeding, organ impairment, and blood clots [4,6].

In order to overcome the undesirable side effects of modern treatment, an external supply of alternative medicine may be necessary to protect the organ from harmful effects. Natural products, including traditional herbs, have a tremendous role in treating diseases, including cancer [7]. Aromatic plants produce essential oils (EOs) as a complex mixture of compounds belonging to secondary metabolites, which are generally obtained through steam or hydrodistillation, solvent extraction, supercritical fluid, and subcritical water extractions [8]. EOs are natural volatile liquids and consist of complex compounds that are characterized by a strong odor. In nature, EOs protect plants from microbes, viruses, fungi, insects, and herbivores by reducing their appetite through the smell of plants [9]. In addition, EOs attract some insects to help with pollen and seed dispersion [9]. This review explores various EOs in the treatment of cervical cancer.

## 2. Literature Research Methodology

This narrative review is composed of a collection of available research studies conducted on the role of EOs in cervical cancer treatment, which includes publications from 2015 to 2023. Electronic databases were queried, including PubMed, Scopus, and Ovid. The following keywords were used individually and in combination as inclusion criteria for articles to be considered for this review: essential oil, natural product, terpene, terpenoid, medicinal herb, medicinal plant, antioxidant, cervical cancer, human papillomavirus, and HPV. Research studies that were (1) non-English full-text articles, (2) non-peer-reviewed articles, (3) irrelevant to the subject matter, (4) reviews, case reports/case series, (5) letters to editors, (6) non-original research articles, (7) conference abstracts, and (8) preprints were excluded from this review. All relevant articles were screened thoroughly by at least two authors. Following the inclusion and exclusion criteria, 74 articles were shortlisted for eligibility.

## 3. Human Papillomavirus as Risk Factors of Cervical Cancer

A strong association between HPV and cervical squamous cell cancer has been reported since the early 1980s [10,11]. HPV is a member of the Papillomaviridae family and one of the most prevalent viral infections that can lead to sexually transmitted diseases (STD) globally [12]. Out of the 200 HPVs identified, a total of 15 genotypes are known to be associated with the development of cervical cancer [13]. HPV types can be further divided into high-, probably high-, and low-risk groups based on their carcinogenic properties. The high-risk group includes HPV-16, -18, -31, -33, -35, -39, -45, -51, -52, -56, -58, -59, -68, -73, and -82, whereby HPV-16 and -18 cause about 70% of all invasive cervical cancers [13,14]. The risk of developing cervical cancer is strongly associated with the persistence of infection, which is more commonly observed in individuals infected with high-risk oncogenic types of HPV. The primary immune response to HPV infection involves the activation of cells. Thus, individuals with conditions that weaken cell-mediated immunity, such as human immunodeficiency virus (HIV) disease or having undergone renal transplantation, have an elevated risk of acquiring and progressing HPV infection [15]. Additionally, *Chlamydia trachomatis* infection was reported to be significantly associated with the development of cervical cancer associated with an HPV infection [16]. Apart from cervical cancer, HPV infection has also been associated with a significant percentage of anogenital (anus, vulva, vagina, and penis), oral cavity, laryngeal, and head and neck cancers [17,18]. Thus, HPV vaccination aimed at reducing morbidity and mortality from HPV-associated disease was introduced in 2006 and has shown high effectiveness against cervical cancer among girls vaccinated younger than 20 years old [19].

## 4. Conventional Treatment of Cervical Cancer

The type and stage of the cancer, potential side effects, the patient’s preferences, and general health are all factors that influence how cervical cancer is treated. Although early-stage cervical cancer can typically be cured with surgery, chemotherapy, radiation therapy, or a combination of these treatments, advanced cervical cancer is frequently incurable and, once recurring, is generally resistant to treatment [20].

The role of minimally invasive surgery (including both laparoscopic and robotic surgery), which offers benefits such as lower operative morbidity and a shorter hospital stay compared to open surgery, like a radical hysterectomy or radical trachelectomy with pelvic lymphadenectomy, has become the standard surgical treatment for early-stage cervical cancer over the past 20 years [1]. Different perspectives on cervical cancer treatment are based on the use of immunotherapy, target therapy, and poly-ADP-ribose polymerases (PARP) inhibitors alone, in combination with conventional chemotherapy, and in combination with radiotherapy [1]. In 2018, pembrolizumab was a type of immune checkpoint inhibitor drug that received FDA approval for PD-L1-positive metastatic or recurrent cervical cancer [21]. Currently, integrative oncology, which combines allopathic and current biological cancer treatments with complementary and alternative medicine therapies, helps in treating cervical cancer [22].

Additionally, the available therapies for cervical cancer are associated with disabling side effects and tumor drug resistance [23,24,25,26]. Patients who undergo more invasive surgery, particularly, radiotherapy, report higher levels of chronic bowel, sexual, and bladder dysfunction [27,28]. After the first and second cycles of concurrent chemoradiotherapy, patients under the age of 60 would substantially more frequently have symptoms of nausea and vomiting [29]. This implies that having this side effect causes a “valuation shift” or higher values for adverse health states [30], as shown in Table 1. The paradigm has evolved throughout time as a result of these side effects of cancer treatment, with a greater focus currently being placed on prevention than on therapy.

## 5. Essential Oils: Composition and Their Potential Benefits

Currently, the use of EOs is focused more on the food, biomedicine, and agricultural industries. EOs extracted from medicinal plants have been reported to possess various medicinal properties, including protection from organ toxicity, acetylcholinesterase activity, and antibacterial and antioxidant properties [31,32,33]. EOs consist of tens to hundreds of different chemical mixtures, such as hydrocarbons, terpenes, aldehydes, alcohols, and organic acids [34]. Their composition is highly diverse across different plant species.

**Figure 1:**
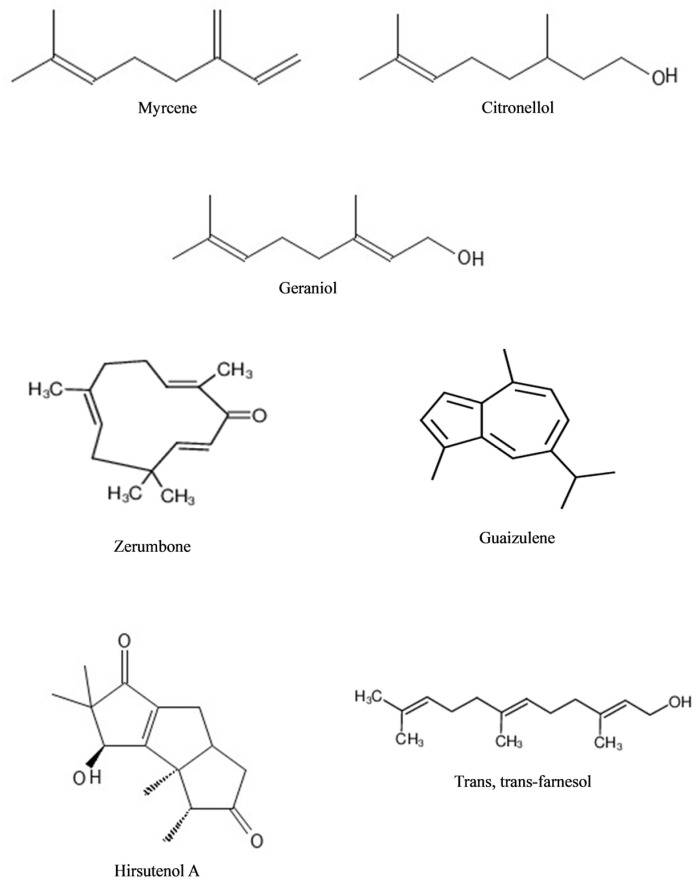
Representative examples of terpenes and terpenoids.

The main compounds of EOs have carbon and hydrogen as their building blocks, of which isoprene (2-methylbuta-1,3-diene, CH_2_=C(CH_3_)CH=CH_2_) is the major basic hydrocarbon unit found in EOs [35]. Isoprene units are joined to form terpenes, which are further classified into monoterpenes, sesquiterpenes, and diterpenes, according to the number of isoprene units in their structure. Monoterpenes are formed by two isoprene units, sesquiterpenes by three units, and diterpenes by four units [35,36]. Limonene, carvone, bisabolol, thymoquinone, terpinen-4-ol, pulegone, isopulegol, geraniol, linalool, citronellol, citronellal, myrcene, thujane, farnesene, azulene, cadinene, and cymene are examples of terpenes that have been widely tested for their medicinal properties [37,38,39,40,41,42,43,44,45,46,47,48,49,50,51,52,53]. Monoterpenes are often the major compounds found in EOs, which may account for about 90% of EO components, followed by sesquiterpenes [54]. Additionally, a small amount of diterpenes, triterpenes, and tetraterpenes, and their oxygenated derivatives are also present in EOs [55]. Terpenoids, another class of terpenes, are produced by removing or adding methyl groups, which consist of oxygen molecules [56]. Terpenoids can be further divided into alcohol, aldehydes, esters, ethers, epoxides, ketones, and phenols. Figure 1 shows the selected structure of terpenes and terpenoids.

Alcohol is converted to aldehydes through a process known as dehydrogenation, which removes the hydrogen atom [57]. Citral, myrtenal, benzaldehyde, and cinnamaldehyde are among the aldehydes present in EOs [44,58]. Generally, aldehydes are volatile and easily oxidized. Most aldehydes are skin sensitizers and mucous membrane irritants [59]. Aldehydes have pleasant fruity odors and are sweet, and they are found in culinary herbs such as cumin and cinnamon [60]. Aldehydes have been reported to possess antipyretic, spasmolytic, calming, vasodilator, antiviral, tonic, and antimicrobial activities [35]. 

Alcoholic terpenoids are found to be spasmolytic, antiseptic, and antimicrobial [61]. Examples of alcohols in EOs are linalool, borneol, nerol, citronellol, and geraniol [56]. Organic acids contain the carboxyl group and are rare in EO. For example, ethanoic acid is produced in vinegar and certain aromatic waters. Acids are important to produce esters. Esters in EOs are produced during the distillation process [59].

## 6. Role of Essential Oils in Cervical Cancer: Evidence from Laboratory

In vitro and animal studies have contributed to our understanding of the potential effects of EOs on cervical cancer and the mechanisms underlying them (summarized in Table 2). According to the American National Cancer Institute, EOs that exhibit cytotoxicity with an IC_50_ < 30 μg/mL may be useful as anticancer agents [62]. Recent studies have mostly investigated the impact of various EOs on human cervical carcinoma cell lines, SiHa (HPV-16) and HeLa (HPV-18). EOs extracted from Turmeric (*Curcuma* sp.) [63,64,65], fennel (*Foeniculum vulgare*) [66,67,68], catmint (*Nepeta* sp.) [69,70,71], sage (*Salvia* sp.) [72,73], thyme (*Thymus* sp.) [74,75], ginger (*Zingiber* sp.) [63,64,76,77], *Moringa* sp. [78,79], *Cinnamomum zeylanicum* [80], *Ephedra intermedia* [81], *Inula graveolens* [82], and *Perilla frutescens* [83] have all shown cytotoxic effects to SiHa and HeLa cell lines. An animal study by Azadi et al. [84] examined the effects of intraperitoneal administration of *Zataria multiflora* EO on tumor growth in a cervical cancer xenograft C57BL/6 mouse model. The findings revealed a significant reduction in tumor volume and angiogenesis, suggesting potential anticancer properties of the EO.

Studies suggested that EOs had inhibitory effects on cell proliferation and induced apoptosis in cancer cell lines [85,86]. For example, Moirangthem et al. [87] reported that *Cephalotaxus griffithii* EO treatment induced cytoplasmic membrane blebbing, nuclear contraction, nuclear fragmentation, and the formation of apoptotic bodies in HeLa cells, associated with up-regulated expression of caspase-3, a central protein involved in the process of apoptosis. Similarly, *Erigeron canadensis* EO treatment led to increased expression of caspase-3, -9, and -12 proteins, nuclear pyknosis, and abnormal chromatin condensation in HeLa cells [88]. Additionally, EOs have been shown to induce cell cycle arrest by blocking cells in the G0/G1 phase [89,90,91].

As described in the previous section, EOs contain various phytochemicals, i.e., hydrocarbons, terpenes, aldehydes, alcohols, and organic acids. As this review has shown, using EOs from the whole plant or a specific portion of the plant (e.g., leaves, stems, flowers, or roots) may prove beneficial in inhibiting cancer cell proliferation. Numerous phytochemicals found in EOs have the potential to operate singly or in combination to treat cervical cancer through a polypharmacy effect [92]. However, identifying and isolating an active phytochemical should also be crucial, particularly during the drug development process.

### 6.1. Essential Oils with High Monoterpenes

Monoterpenes are composed of two isoprene units, which are 5-carbon building blocks connected to form a 10-carbon structure. The general chemical formula for a monoterpene is C_10_H_16_ [93]. Monoterpenes can have different structural arrangements, leading to a wide variety of compounds within this class [94]. α-Pinene, limonene, and sabinene are monoterpenes that form the major constituents of *Juniperus communis* EO, and have displayed cytotoxicity against SiHa cells compared to vinblastine at a dose of 200 μg/mL [95]. Similarly, EOs of *Alpinia nigra* (contains 56.27% β-pinene), *Ganoderma applanatum* (contains 23.6% d-limonene), *Lantana camara* (contains 20.38% sabinene), and *Pistacia lentiscus* (contains 56.2% α-pinene) also reported reduced cell viability of cancer cell lines [96,97,98,99]. *Artemisia arborescens* EO contains abundant monoterpene β-thujone (79.16–89.64%) and was shown to reduce the cell viability of HeLa cells with an IC_50_ of 326–467 μg/mL [100]. α-Thujone is also the main constituent (13.5%) found in the leaves and flowers of *Ferula tingitana* EO. It demonstrates a high cytotoxic effect against HeLa cells compared to doxorubicin, with an IC_50_ 4.7 μg/mL [101]. *Aegle marmelos* EO demonstrates a cytotoxic potential at IC_50_ of 85.6 μg/mL against HeLa cells due to its major compounds, p-mentha-1,4(8)-diene (33.2%) and limonene (13.1%) [102]. Other herbal EOs that contain monoterpenes as their major constituents, such as *Aloysia citriodora* (α-citral 43.46–47.62%), *Crassocephalum crepidioides* (β-myrcene 65.9%), and *Litsea cubeba* (geranial 37.67%), reported cytotoxicity to HeLa cells [103,104,105].

### 6.2. Essential Oils with High Sesquiterpenes

Generally, sesquiterpenes are discovered in higher plants, invertebrates, and fungi. They consist of an acyclic, mono-, bi-, tri-, and tetracyclic structure [106]. Sesquiterpenes undergo biotransformation in the organism, which involves two phases: Phase I (sesquiterpenes are converted through oxidation, reduction, or hydrolysis, which may lead to the formation of different metabolites); and Phase II (metabolites from Phase I are conjugated with certain endogenous compounds, e.g., glucuronic acid, glutathione, amino acids, and sulfate). For example, the biotransformation of β-caryophyllene yielded a major metabolite resulting from region- and stereo-selective hydroxylation of the methyl group at carbon 14 and epoxidation at carbon 5/carbon 6 [107]. β-Elemene (IC_50_ 17 µg/mL), bicyclogermacrene (IC_50_ 12.4 µg/mL), (E)-caryophyllene (IC_50_ 10 µg/mL), and germacrene D (IC_50_ 14.5 µg/mL), which are the major constituents of *Piper cernuum* EO, demonstrated more potent anticancer activity in HeLa cells compared to *P. cernuum* EO (IC_50_ 23 µg/mL) [108]. *Piper regnelli* and *Pinus eldarica* EOs contain a high amount of sesquiterpene (germacrene D and β-caryophyllene) [109,110]. Both germacrene D and β-caryophyllene displayed higher cytotoxic potential compared to cisplatin [109]. *Nectandra leucantha* EO displayed significant cytotoxic activity against HeLa cells (IC_50_ 60 μg/mL), with the main identified compound, bicyclogermacrene (28.44%), displaying an IC_50_ ranging from 3.1 to 21 μg/mL [111]. Similarly, *Mikania micrantha* EO containing isoledene (16%) as its major constituent reported cytotoxicity to HeLa cells with an IC_50_ of 5.44 μg/mL [112].

### 6.3. Essential Oils with High Monoterpenoids

Treatment with EOs of *Dittrichia viscosa*, *Hedychium coronarium*, and *Rosmarinus officinalis* with monoterpenoid 1,8-cineole as the major constituent reported cytotoxicity effects to HeLa cells [63,113,114]. *Lavandula pubescens*, *Origanum acutidens,* and *Satureja boissieri* EOs contain high amounts of carvacrol, a monoterpenoid compound [115,116,117]. These EOs exhibited a significant cytotoxic effect against HeLa cell lines at tested concentrations. EOs from *Cymbopogon nardus* (containing 33.06% citronellal), *Mentha piperita* (containing 43.9% menthol), and *Syzygium aromaticum* (containing 85.2% eugenol) were also reported to reduce the viability of HeLa cells [74,118]. *trans*-Piperitol represents 51.2% of *Peucedanum dhana* EO [119]. Treatment with *trans*-piperitol in HeLa cells reported reduced cell viability with an IC_50_ of 7.07 μg/mL, demonstrating a significantly higher anticancer property compared to *P. dhana* EO (IC_50_ 56.63 μg/mL).

### 6.4. Essential Oils with High Sesquiterpenoids

*Siegesbeckia pubescens* EO was dominated by oxygenated sesquiterpenes (83.46%), oxygenated diterpenes (3.69%), and hydrocarbon sesquiterpenes (0.94%), with caryophyllene oxide (21.89%), germacra-4(15),5E,10(14)-trien-1β-ol (14.10%), trans-longipinocarveol (5.87%), (−)-spathulenol (5.14%), and dehydrosaussurea lactone (4.85%) as the main components [120]. It exhibited strong cytotoxicity with an IC_50_ of 37.72 μg/mL for HeLa cell lines. In addition, *S. pubescens* EO is also able to reduce NO release by LPS-induced RAW264.7 macrophages [120]. *Acorus calamus* EO contains β-asarone as its major constituent. The cytotoxic activity of *A. calamus* EO was recorded to inhibit the proliferation of SiHa cells, ranging from 55.5 to 74.9%, after exposure to 300 μg/mL EO [121].

**Table 2:** Summary of essential oil in treating cervical cancer in preclinical research.

Essential Oil	Major Constituent	Study Model, Treatment Regimen	Important Findings	References
*Achillea millefolium* (Yarrow)	1,8-Cineole (27.3%) Camphor (24.3%) β-Eudesmol (18.7%)	HeLa human cervical epithelioid carcinoma cells	Reduced cell viability (IC_50_ ND). Blocked cells in G0/G1 phase.	[89]
*Acorus calamus* (Sweet flag)	β-Asarone (31.56–91.27%) α-Asarone (1.05–52.96%)	SiHa human cervical cancer cells	Reduced cell viability (IC_50_ 55.5%) at 300 µg/mL.	[121]
*Aegle marmelos* (L.) Correa (Bael tree)	p-Mentha-1,4(8)-diene (33.2%) Limonene (13.1%) p-Cymen-α-ol (9.5%) γ-Gurjunene- (7.9%) β-Phellandrene (4.3%)	HeLa cells	Reduced cell viability (IC_50_ 85.6 μg/mL). Effective in suppressing ROS.	[102]
*Aloysia citriodora* (Lemon verbena)	α-Citral (43.46–47.62%) α-Curcumene (11.35–14.39%) trans-1,2-Bis-(1-methylethenyl)cyclobutane (10.08–15.07%)	HeLa cells	Reduced cell viability (IC_50_ 84.5 and 33.31 μg/mL, vs. IC_50_ 22.01 μg/mL (Doxorubicin)). Inhibited COX-1 and COX-2 enzymes.	[103]
*Alpinia nigra* (Gaertn.) Burtt (Black Galangal)	Leaves: β-pinene (56.27%) α-Farnesene (7.92%) Caryophyllene (6.46%) Rhizomes: β-pinene (38.03%) myrtenol (9.35%) α-Humulene (7.82%) Humulene epoxide II (6.00%)	HeLa cells	Inhibited 60% (leaves EO) and 79% (rhizomes EO) proliferations at 20 µg/mL.	[96]
*Artemisia arborescens* (Vaill.) L. (Silver wormwood)	β-Thujone (79.16–89.64%) Camphor (5.34–6.58%) β-Pinene (2.01%) Sabinene (3.44%)	HeLa cells	Reduced cell viability (IC_50_ 326 and 467 μg/mL). Inhibit COX-1 and COX-2.	[100]
*Cephalotaxus griffithii* Hook. f. (Griffith’s plum yew)	ND	HeLa cells	Reduced cell viability (IC_50_ ND). Induced apoptosis (up-regulated caspase-3 expression, reduced cell volume, increased cytoplasmic membrane blebbing, nuclear contraction, nuclear fragmentation, and formation of apoptotic bodies). Inhibit cell migration.	[87]
*Chenopodium botrys* L. (Jerusalem-oak)	α-Eudesmol (16.81%) Elemol acetate (13.2%) Elemol (9.0%) α-Chenopodiol-6-acetate (7.9%)	HeLa cells	Reduced cell viability (IC_50_ 79.62 µg/mL). Increased numbers of apoptotic cells. Induced cell cycle arrest in G1 phase. Increased *p21*, *p53*, *Bax,* and *caspase-3* expressions.	[90]
*Cinnamomum zeylanicum* Blume (Cinnamon)	Cinnamaldehyde (77.34%) *trans*-Cinnamyl acetate (4.98%) Benzene dicarboxylic acid (3.55%) α-Pinene (2.6%)	HeLa cells	Reduced cell viability (IC_50_ 0.13 μg/mL).	[80]
*Crassocephalum crepidioides* (Thickhead weed)	β-Myrcene (65.9%) β-Phellandrene (8.8%) α-Pinene (3.1%) α-Copaene (1.5%)	SiHa cells	Reduced cell viability (IC_50_ 45.9 µg/mL).	[104]
*Curcuma aromatica* (Wild turmeric)	ar-Tumerone (ND)	HeLa cells	Reduced cell viability (IC_50_ 72.02 µg/mL).	[63]
*C. longa* L. (Turmeric)	ar-Tumerone (ND)	HeLa cells	Reduced cell viability (IC_50_ 24.82 µg/mL).	[63]
	ar-Turmerone (33.2%) α-Turmerone (23.5%) β-Turmerone (22.7%)	HeLa cells	Reduced cell viability (IC_50_ 36.6 µg/mL). HeLa cell morphology showed condensation of chromatin, loss of cell membrane integrity with protrusions, and cell content leakage.	[64]
*C. zedoaria* (Christm.) Roscoe (White turmeric)	Zerumbone (17.2%) Camphor (17.56%) Curzerenone (10.2%) Isovelleral (6.6%)	HeLa and SiHa cells	Reduced HeLa cell viability (IC_50_ 6.4 μg/mL vs. IC_50_ 6.5 μg/mL (doxorubicin)). Reduced SiHa cell viability (IC_50_ 9.8 μg/mL vs. IC_50_ 7.8 μg/mL (doxorubicin)).	[65]
*Cymbopogon nardus* (Citronella grass)	Citronellal (33.06%) Geraniol (28.40%) Nerol (10.94%) Elemol (5.25%)	HeLa cells	Reduced cell viability (IC_50_ 142 μg/mL).	[118]
*Dittrichia viscosa* (L.) Greuter (Yellow fleabane)	1,8-Cineole (16.41%) Caryophyllene oxide (15.14%) α-Terpinyl acetate (13.92) α-Muurolol (13.75%)	HeLa cells	Reduced cell viability (IC_50_ 660 μg/mL).	[113]
*Ephedra intermedia* Schrenk and Mey	2-Ethyl-pyrazine (67.37%) γ-Elemene (9.21%) Benzyl acetate (9.10%) 2-Methyl-butyl acetate (5.28%)	HeLa cells	Reduced cell viability (IC_50_ 423.22 μg/mL).	[81]
*Erigeron canadensis* L. (Canadian horseweed)	Limonene (65.68%) (Z)-β-ocimene (6.87%) β-Pinene (6.29%) Germacrene (4.03%)	HeLa cells	Reduced cell viability (IC_50_ 6780 μg/mL vs IC_50_ 1120 μg/mL (Limonene alone)). Cells showed nuclear pyknosis and abnormal chromatin condensation after EO treatment. Decreased G1 phase cells and increased G2/M phase cells. Increased expression of Caspase-3, -9, and -12 proteins. Inhibited mitochondrial membrane potential.	[88]
*Ferula tingitana* (L.) Apiaceae (Giant Tangier fennel)	Leaves: δ-Cadinol (13.8%) γ-Eudesmol (9.7%) 7-α-Eudesma-3,5-diene (9.0%) Elemol (8.3%) Fruit^s^: 3-Carene (13.9%) α-Thujene (13.5%) Elemol (8.9%) Myrcene (8.1%)	HeLa cells	Reduced cell viability (IC_50_ 10.9 µg/mL (leaves EO), IC_50_ 8.6 µg/mL (fruits EO) vs. IC_50_ 4.7 µg/mL (doxorubicin)).	[101]
*Foeniculum vulgare* (Fennel)	*trans*-Anethole (36.8%) p-Anisaldehyde (7.7%) α-Ehyl-p-methoxybenzyl alcohol (9.1%)	HeLa cells	Reduced cell viability (IC_50_ 207 mg/L).	[66]
	*trans*-Anethole (80.63%) L-Fenchone (11.57%) Estragole (3.67%) Limonene (2.68%)	HeLa cells	Reduced cell viability (IC_50_ 1.26 μg/mL).	[67]
	*trans*-Anethole (88.28%) Estragole (4.25%) D-Limonene (2.04%) Fenchone (2.03%)	HeLa cells	Reduced cell viability (IC_50_ 56.43 µg/mL vs. 17.95 µg/mL (Paclitaxel)).	[68]
*Ganoderma applanatum* (Artist’s conk)	γ-Terpinene (30.3%) d-Limonene (23.6%) Cis-2-methyl-4-pentylthiane-s,s-dioxide (15.3%) Cymene (12.7%)	HEp-2 cervical cancer cells	Reduced cell viability (IC_50_ 43.2 μg/mL).	[97]
*Hedychium coronarium* (White ginger lily)	1,8-Cineole (ND)	HeLa cells	Reduced cell viability (IC_50_ 87.98 µg/mL).	[63]
*Helichrysum italicum* (Roth) G. Don (Curry plant)	α-pinene (21.6%) γ-curcumene (21.6%) Neryl acetate (7.9%)	HeLa cells	Reduced cell viability (IC_50_ 7.5 µg/mL). No effect on cell cycle. Induced apoptosis.	[85]
*Hyptis suaveolens* (L.) Poit. (Pignut)	Sabinene (14.03%) Eucalyptol (12.78%) β-Caryophyllene (11.27%) Bicyclogermacrene (8.08%)	HeLa cells	Reduced cell viability (IC_50_ 181.37 µg/mL vs. IC_50_ 4.32 (cisplatin)). Induced G0/G1 cell cycle arrest and a decreased G2/M phase.	[91]
*Inula graveolens* (Linnaeus) Desf (Stinkwort)	Bornyl acetate (69.15%) Camphene (11.11%)	HeLa cells	Reduced cell viability (IC_50_ 64.1 µg/mL, IC_50_ 72.0 µg/mL (bornyl acetate) vs. IC_50_ 126.75 µg/mL (cisplatin)).	[82]
*Juniperus communis* (Common Juniper)	α-Pinene, limonene, and sabinene (49.1–82.8%) 4-Terpineol	SiHa cells	Reduced cell viability (IC_50_ 150.6 µg/mL).	[95]
*Kaempferia galanga* (Sand ginger)	Ethyl p-methoxycinnamate (ND)	HeLa cells	Reduced cell viability (IC_50_ 44.18 µg/mL).	[63]
*Lantana camara* Linn. (Common Lantana)	Sabinene (20.38%) β-Caryophyllene (17.88%) Eucalyptol (10.56%) α-Humulene (6.68%)	HeLa cells	Reduced cell viability (IC_50_ 229.27 µg/mL).	[98]
*Lavandula pubescens* Decne. (Downy Lavender)	Carvacrol (72.7%) Carvacrol methyl ether (7.0%) Caryophyllene oxide (5.9%)	HeLa cells	Reduced cell viability (IC_50_ < 10 µg/mL).	[117]
*Litsea cubeba* (Mountain pepper)	Geranial (37.67%) Neral (32.75%) Limonene (10.55%)	HeLa cells	Reduced cell viability (IC_50_ 67.7 µg/mL).	[105]
*Melaleuca alternifolia* (Maiden and Betche) Cheel (Tea tree)	Terpinen-4-ol (41.9%) γ-Terpinene (17.8%) α-Terpinene (8%) p-Cymene (4.6%)	HeLa cells	Reduced cell viability (IC_50_ ND).	[74]
*Mentha piperita* L. (Peppermint)	Menthol (43.9%) Menthone (23.1%) 1,8-Cineole (6.6%) Menthyl acetate (4.9%)	HeLa cells	Reduced cell viability (IC_50_ ND).	[74]
*Mikania micrantha* Kunth (Bittervine)	Isoledene (16%) δ-Cadinene (11.2%) Debromofiliformin (9.4%) trans-Caryophyllene (9.1%)	HeLa cells	Reduced cell viability (IC_50_ 5.44 μg/mL).	[112]
*Moringa oleifera* Lam. (Horseradish tree)	ND	HeLa cells	Reduced cell viability (IC_50_ 422.8 µg/mL).	[78]
*Moringa peregrina* (Ben tree)	ND	HeLa cells	Reduced cell viability (IC_50_ 366.3 µg/mL).	[79]
*Nectandra leucantha* Nees and Mart.	Bicyclogermacrene (28.44%) Germacrene A (7.34%) Spathulenol (5.82%) Globulol (5.25%)	HeLa & SiHa cells	Reduced cell viability (IC_50_ 60 µg/mL, 12.4 µg/mL (Bicyclogermacrene), vs. 20 µg/mL (cisplatin)).	[111]
*Nepeta curviflora* Boiss (Syrian catmint)	1,6-Dimethyl spiro [4.5] decane (27.5%) Caryophyllene oxide (20.1%) β-caryophyllene (18.3%)	HeLa cells	Reduced cell viability (IC_50_ 746.9 and 453.1 µg/mL). Inhibit cell migration.	[69]
*Nepeta rtanjensis* Diklić and Milojević (Rtanj catmint)	*trans,cis*-Nepetalactone (71.66%) *cis,trans*-Nepetalactone (17.21%) α-Pinene (3.28%)	HeLa cells	Reduced cell viability (IC_50_ 0.050 μL/mL). Cells demonstrated cytoplasmic shrinkage and nuclear condensation and fragmentation, cell membrane blebbing, and occurrence of apoptotic bodies. Induced cell cycle perturbations. Increased number of cells with fragmented DNA. Up-regulated *Bax* and *p53* expressions. Down-regulated *Bcl-2* and *Skp2* expressions.	[70]
*Nepeta sintenisii* Bornm.	4aα,7α,7aβ-Nepetalactone (51.74%) β-Farnesene (12.26%) 4aα,7α,7aα-Nepetalactone (8.01%) Germacrene-D (5.01%)	HeLa cells	Reduced cell viability (IC_50_ 20.37 µg/mL).	[71]
*Origanum acutidens* (Hand-Mazz.) Ietswaart	Carvacrol (61.69%) p-Cymene (17.32%) γ-Terpinene (4.05%) Borneol (3.96%)	HeLa cells	Reduced cell viability (IC_50_ < 10 µg/mL).	[115]
*Perilla frutescens* (L.) Britt.	Perilla ketone (80.88%) Apiol (1.77%) β-Caryophyllene (1.59%)	HeLa cells	Reduced cell viability (IC_50_ 34.58 μg/mL).	[83]
*Peucedanum dhana* A. Ham	trans-Piperitol (51.2%) o-Cymene (11.1%) γ-Terpinene (9.2%)	HeLa cells	Reduced cell viability (IC_50_ 56.63 μg/mL vs 7.07 μg/mL (trans-piperitol)).	[119]
*Pinus eldarica* (Eldar pine)	β-Caryophyllene (14.8%) Germacrene D (12.9%) α-Terpinenyl acetate (8.15%) α-Pinene (5.7%)	HeLa cells	Reduced cell viability (IC_50_ ND).	[110]
*Pistacia lentiscus* var. *chia* (Mastic tree)	Wild plant: α-Pinene (56.2%, 51.9%) Myrcene (20.1%, 18.6%) β-Pinene (2.7%, 3.1%) Cultivated plant: α-Pinene (70.8%) β-Pinene (5.7%) Myrcene (2.5%)	HeLa cells	Reduced cell viability (IC_50_ 20.11–18.81 µg/mL, IC_50_ 7.62 µg/mL vs. IC_50_ 2.14 µg/mL (Doxorubicin)).	[99]
*Piper cernuum* Vell. (Pariparoba)	β-Elemene (30.0%) Bicyclogermacrene (19.9%) (E)-Caryophyllene (16.3%) Germacrene D (12.7%)	HeLa cells	Reduced cell viability (IC_50_ 23 µg/mL, 17 µg/mL (β-elemene), 12.4 µg/mL (bicyclogermacrene), 10 µg/mL ((E)-caryophyllene), 14.5 µg/mL (germacrene D))	[108]
*Piper regnellii* (Miq) C. DC. var. *regnellii* (C. DC.) Yunck	Germacrene D (45.6–51.4%) α-Chamigrene (8.9–11.3%) β-Caryophyllene (8.2–9.5%)	HeLa cells	Reduced cell viability (IC_50_ 7 µg/mL (germacrene D), 11 µg/mL (α-chamigrene), 32 µg/mL (β-caryophyllene) vs. 20 µg/mL (cisplatin)).	[109]
*Rosmarinus officinalis* L. (Rosemary)	1,8-Cineole (23.56%) Camphene (12.78%) Camphor (12.55%) β-pinene (12.3%)	HeLa cells	Reduced cell viability (IC_50_ 0.011 µg/mL).	[114]
*Salvia officinalis* L. (Garden sage)	α-Thujone (ND) 1,8-Cineole (ND) Camphor (ND)	HeLa cells	Reduced cell viability (IC_50_ ND).	[72]
*Salvia sclarea* L. (Clary sage)	ND	HeLa cells	Reduced cell viability (IC_50_ 80.69 μg/mL). Cells demonstrated apoptotic bodies, e.g., blebbing, cell breakage, and chromatin condensation.	[73]
*Satureja boissieri* Hausskn. Ex Boiss. (Catri/Kekik)	p-Cymene (23.15%) γ-Terpinene (22.84%) Carvacrol (21.25%) Thymol (18.96%)	HeLa cells	p-Cymene, thymol, and carvacrol inhibited cell viability.	[116]
*Siegesbeckia pubescens*	Caryophyllene oxide (21.89%) trans-longipinocarveol (5.87%) dehydrosaussurea lactone (4.85%)	HeLa cells	Reduced cell viability (IC_50_ 37.72 μg/mL).	[120]
*Syzygium aromaticum* (L.) Merr. and L.M. Perry (Clove]	Eugenol (85.2%) (E)-β-Caryophyllene (9.9%)	HeLa cells	Reduced cell viability (IC_50_ ND).	[74]
*Tagetes ostenii* Hicken	Dihydro-tagetone (64.2%) (E)-Ocimenone (39.9%) (Z)-β-Ocimene (26.1%) (Z)-Ocimenone (17.5%)	SiHa cells	Reduced cell viability (IC_50_ 0.072–0.083 µg/mL). Inhibit the adhesion process and clonogenic ability after 24 h of treatment.	[86]
*Thymus vulgaris* L. (Thyme)	Thymol (48.6%) p-Cymene (18.4%) γ-Terpinene (8.8%) Carvacrol (5.5%)	HeLa cells	Reduced cell viability (IC_50_ ND).	[74]
*Thymus bovei* Benth. (Thyme)	Geraniol (32.3%) α-Citral (27.7%) β-Citral (12.4%) Thymol (3.8%)	HeLa cells	Reduced cell viability (IC_50_ 7.22 µg/mL vs. IC_50_ 4.24 µg/mL (cisplatin)).	[75]
*Zataria multiflora* (Shirazi thyme)	Carvacrol (52.2%) γ-Terpinene (12.4%) Carvacrol methyl ether (10.23%) p-Cymen (4.3%)	TC1 mouse cervical cancer cells	Reduced cell viability (IC_50_ 15.62–66.52 µg/mL). Promoted apoptosis through caspase-3 dependent pathway.	[84]
		Subcutaneous inoculation of TC1 cells (8 × 10^5^) in C57BL/6 mice; 500 mg/kg, 7 days, intraperitoneally	Decreased tumor weight. Increased secretion of TNF-α, IFN-γ, and IL-2. Decreased secretion of IL-4.	
*Zingiber officinale* Roscoe (Ginger)	α-Zingiberene (35%) ar-Curcumene (15.3%) β-Sesquiphellandrene (12.3%)	SiHa cells	Reduced cell viability (IC_50_ 38.6 µg/mL and 46.2 µg/mL). Produced nucleosomal DNA fragmentation. Increased cytochrome C release and caspase-3 activation.	[76]
	Camphene (16.4%) Geranial (9.9%) 1.8-Cineole (8.9%) β-Phellandrene (8.8%)	HeLa cells	Reduced cell viability (IC_50_ 129.9 µg/mL). HeLa cell morphology showed condensation of chromatin, loss of cell membrane integrity with protrusions, and cell content leakage.	[64]
*Zingiber ottensii* (Malaysian Ginger)	ND	HeLa cells	Reduced cell viability (IC_50_ 1:3000 dilutions). Induced apoptosis. Activated intrinsic apoptotic pathway via caspase and PARP pathway. Decreased IL-6 in a dilution-dependent manner.	[77]

EO, essential oil; IC_50_, half maximal inhibitory concentration; IFN-γ, interferon gamma; IL, interleukin; ND, not defined; TNF-α, tumor necrosis factor-alpha.

### 6.5. Essential Oils Clinical Trials in Cervical Cancer and HPV Infection

Compared to the extensive studies reporting the anticancer activity of EOs in cervical cancer cells, there is a lack of comprehensive clinical data. Nevertheless, some clinical studies and trials have been conducted to investigate the potential role of EOs in cervical cancer management and the treatment of HPV infection. These studies have provided valuable insights into the efficacy and safety of EOs as a complementary therapy. Here, we summarize some key clinical findings (Table 3).

Blackburn et al. [122] conducted a randomized controlled trial of 41 locally advanced cervical cancer patients who received intracavitary brachytherapy to investigate the effect of foot reflexology and aromatherapy on anxiety and pain. The results revealed a significant reduction in average pain and anxiety scores, indicating the potential benefits of foot reflexology and aromatherapy for emotional support during cancer treatment. Similarly, Sriningsih et al. [123] also reported improved chemotherapy-induced nausea and vomiting following ginger aromatherapy in post-cervical cancer chemotherapy patients.

The effect of an herbal vaginal suppository containing 10% *Myrtus communis* L. aqueous extract and 0.5% EO was tested in a randomized double-blind placebo trial of 60 patients with cervicovaginal HPV infection [124]. Results showed that there was a significant increase in HPV test negative results and reduced cervical lesion size in the intervention group compared to the control group, suggesting that a *M. communis* herbal suppository can speed up virus clearance and may be effective in treating HPV infection. In contrast, topical application with a mixture of natural EOs (eugenol, carvone, nerolidol, and geraniol) in olive oil given to visual inspection of the cervix with acetic-acid-positive patients showed no differences in the regression of the lesion and HPV clearance rate between the intervention and control groups [125].

## 7. Future Directions and Challenges

The body of research on EOs in cervical cancer is continuing to grow, and ongoing research may uncover new EOs and compounds with enhanced therapeutic potential. Considering the potential benefits of EO in inhibiting cancer cell viability, further investigations are required for clinical trials. In this section, we delve into the future direction and challenges of integrating EO with conventional treatments, offering a comprehensive view of the current landscape and the path forward in cervical cancer care.

### 7.1. Innovation in the Delivery of Essential Oils

Although numerous studies have reported the high anticancer properties of EOs in cervical cancer cells, EOs are frequently linked to high volatility, poor stability, and being easily broken down when exposed to oxygen, heat, humidity, or light [126]. Moreover, the poor solubility of EOs in water limits their bioavailability [127]. Innovations in the delivery of EO, e.g., nanoparticle (NP) encapsulation, nanoemulsification, or inclusion with hydrophilic substances, may open up new avenues for the application of EOs in cancer treatment (Table 4).

The poor stability and bioavailability of EOs can be improved by encapsulating them in the form of solid lipid NPs (SLNs) [128]. This technique is based on building nanostructures in which EOs are attached to or enclosed within submicron-sized capsules or NPs that enhance their bioavailability and improve targeted delivery to cancer cells [127]. *Eucalyptus globulus* EO encapsulated to SLNs significantly enhanced the cytotoxic effect against HeLa cells, with an IC_50_ of 21.30 μg/mL, compared to the EO alone (IC_50_ 33.20 μg/mL) [129]. Similarly, encapsulation of EO’s active compounds (e.g., thymoquinone, the main constituent of *Nigella sativa* EO, and eugenol, the main constituent of *S. aromaticum* EO) has been reported to have greater inhibition of HeLa cell viability compared to EOs alone [130,131].

Nanoemulsions are also recognized as an ideal vehicle for delivering lipophilic compounds due to their small particle size, simplicity in manufacture, enhanced bioavailability, biological efficacy, and kinetic stability [132]. Nanoemulsifications of *Rosa damascene* and *Melaleuca alternifolia* EOs have shown great effect in inhibiting HeLa cell viability [133,134]. Furthermore, *M. alternifolia* nanoemulsions were stable under centrifugal, freeze–thaw stress and long-term storage for up to 50 days at different temperatures [134]. Combining nanoemulsions with a gel foundation forms nanoemulgels, which are particularly well suited for topical application [135]. Both *Coriandrum sativum* and *Zingiber ottensii* EOs nanoemulgels have enhanced cytotoxicity to HeLa cells, compared to EO alone [136,137]. However, *Z. ottensii* nanoemulgel (IC_50_ 8.88 μg/mL) reported a lower anticancer effect compared to *Z. ottensii* nanoemulsion (IC_50_ 5.81 μg/mL), most likely due to the retardation effects of the gel component of *Z. ottensii* nanoemulgel, which prolonged the release of *Z. ottensii* EO [137].

Cyclodextrins (CD) are cyclic oligosaccharides composed of (α-1,4)-glucopyrannose units, which have an unusual structure where their interior chamber is hydrophobic, and their external surface is hydrophilic. This property allows inclusion complexes to form with both inorganic and organic materials. β-CD is the most widely used CD since its cavity can hold molecules weighing between 200 and 800 g/mol [138]. The β-CD/*S. aromaticum* inclusion complex (IC_50_ 12.5 µg/mL) demonstrated higher anticancer properties against HeLa cells compared to EO alone (IC_50_ 190.0 µg/mL)[139]. However, it is important to note that the increased anticancer properties of the β-CD/*S. aromaticum* inclusion complex were also associated with increased cytotoxicity against normal VERO cells (IC_50_ 210.0 µg/mL). The β-CD/*Eugenia brejoensis* inclusion complex increased the thermal stability of *E. brejoensis* EO, but also reduced the anticancer activity of *E. brejoensis* EO (CC_50_ 886.71 µg/mL) when compared to EO alone (CC_50_ 63.20 µg/mL) [140]. These results suggested that the features of the β-CD/EO inclusion complexes are influenced by the EO constituent, molecular size, and chemical structure.

**Table 4:** Summary of essential oil in treating cervical cancer in clinical trials.

Essential Oils	Nanocarrier (Particle Size)	Study Model	Important Findings	References
*Eucalyptus globulus* L. (Eucalyptus)	SLN (ND)	HeLa cells	IC_50_ 33.20 μg/mL (EO) IC_50_ 21.30 μg/mL (SLN) IC_50_ 0.24 μg/mL (Doxorubicin)	[129]
Thymoquinone (Main constituent of *Nigella sativa* EO)	SLN (35.66 nm)	HeLa and SiHa cells	SiHa cells: IC_50_ 19.42 (24 h), 10.42 (48 h), 8.50 μg/mL (72 h) HeLa cells: IC_50_ 23.00 (24 h), 18.17 (48 h), 15.58 μg/mL (72 h)	[130]
Eugenol (Main constituent of *Syzygium aromaticum* EO)	NP encapsulation with chitosan (250–351 nm)	HeLa cells	Greater inhibition of cell viability compared to EO (IC_50_ ND). Increased cells in G0/G1 interphase. Decreased cells in G2/M phase.	[131]
*Rosa damascene* (Rose)	Nanoemulsion (30–50 nm)	HeLa cells	IC_50_ 4.6 µg/mL (nanoemulsion)	[133]
*Melaleuca alternifolia* (Tea tree)	Nanoemulsion (300 nm)	HeLa cells	Stable under centrifugal, freeze thaw stress and long-term storage (50 days). Greater inhibition of cell viabilitycompared to paclitaxel (IC_50_ ND).	[134]
*Zingiber ottensii* (Malaysian Ginger)	Nanoemulsion (13.8 nm) Microemulsion (21.2 nm) Nanoemulgel (99.5 nm) Microemugel (99.2 nm)	HeLa cells	IC_50_ 23.25 μg/mL (EO) IC_50_ 5.81 μg/mL (Nanoemulsion) IC_50_ 7.24 μg/mL (Microemulsion) IC_50_ 8.88 μg/mL (Nanoemulgel) IC_50_ 11.88 μg/mL (Microemulgel)	[137]
*Coriandrum sativum* (Cilantro)	Nanoemulgel (<200 nm)	HeLa cells	IC_50_ 67.60 µg/mL (EO) IC_50_ 24.54 µg/mL (Nanoemulgel) IC_50_ 10.11 µg/mL (Doxorubicin)	[136]
*Eugenia brejoensis*	Coprecipitation with β-CD (ND)	HeLa cells	Increased thermal stability of EO. CC_50_ 4460.55 µg/mL (β-CD) CC_50_ 63.20 µg/mL (EO) CC_50_ 886.71 µg/mL (β-CD/EO)	[140]
*Syzygium aromaticum* (Clove)	Kneading with β-CD (ND)	HeLa cells	IC_50_ > 500 µg/mL (β-CD) IC_50_ 190.0 µg/mL (EO) IC_50_ 12.5 µg/mL (β-CD/EO)	[139]

β-CD, β-cyclodextrins; EO, essential oil; IC_50_, half maximal inhibitory concentration; ND, not defined; SLN, solid lipid nanoparticles.

### 7.2. Combining Essential Oils with Conventional Treatments

The integration of EO with conventional drugs in the treatment of cervical cancer aims to harness the potential synergies between the therapeutic properties of EO and current medical interventions, in order to enhance treatment efficacy. Due to its well-established anticancer, anti-inflammatory, and antioxidant properties, EO may complement the results of cancer treatment, e.g., radiation, chemotherapy, or surgery, or soothe their side effects.

Various EOs incorporated with chemotherapeutic agents showed an enhancement in cytotoxicity in cervical cancer cells. Nanoemulsions of cinnamon, peppermint, clove, lemon, salvia, chamomile, frankincense, garlic, and ginger oils loaded with chemotherapeutic agents (e.g., bleomycin, paclitaxel, ifosfamide, mitomycin C, and doxorubicin) presented a major pro-apoptotic capability in HeLa cells compared with the chemotherapeutic agents alone (Table 5) [141,142,143,144,145,146]. Furthermore, peppermint-oil-based microemulsion loaded with paclitaxel was shown to be stable under centrifugal and freeze–thaw stress, and ≈90% of paclitaxel was released in the first 48 h [142]. The combined therapy of monoterpenoid carvacrol-fabricated chitosan NP and doxorubicin, a topoisomerase inhibitor, has also been reported to have greater anticancer effects on HeLa cells with an IC_50_ of 5.66 μg/mL, compared to doxorubicin treatment alone (IC_50_ 6.30 μg/mL)[147]. Thus, the use of EO as an adjuvant with chemotherapeutic agents can be a vital option for cancer treatment, as it will enhance the stability and efficacy of the EO.

### 7.3. Challenges of Using Essential Oils in Cervical Cancer Treatment

The Food and Drug Administration (FDA) has defined some EOs as Generally Recognized as Safe (GRAS) to be used as food additives [148]. For example, *Minthostachys verticillata* EO administered on diet at doses of 0, 1, 4, and 7 g/kg feed to Wistar rats for 90 days showed no mortality, adverse effects on general conditions, or changes in body weight, food consumption, or feed conversion efficiency [149]. However, there is still a lack of safety assessment studies for most EOs in animal models, as well as human studies. As the use of EOs in cervical cancer treatment evolves, it is important to address the safety of EO usage by understanding the potential adverse effects and toxicity of EOs, which are critical for patient safety.

Numerous studies have reported the potential anticancer effects of various EOs on cervical cancer cells. However, it is equally important to examine the cytotoxicity effects of these EOs on normal cells. Furthermore, as mentioned in the previous section, one of the major challenges is the lack of studies that have been performed to examine the effect of EOs on cervical cancer animal models and human patients. The shortage of these studies poses challenges in establishing the efficacy and safety of EOs for cervical cancer treatment.

## 8. Conclusions

Cervical cancer is one of the main killers of women worldwide. Various types of chemotherapeutic drugs have been developed to treat cervical cancer, but most have severe side effects on major organs such as the liver, kidney, and heart. In this review, we explore the anticancer role of various EOs, mostly in vivo against cervical cancer cells. Particularly, EOs from *C. zeylanicum*, *C. zedoaria*, *F. tingitana*, *F. vulgare*, *H. italicum*, *L. pubescens*, *M. micrantha*, *O. acutidens*, *P. regnellii*, *R. officinalis*, *T. ostenii*, and *T. bovei* have proved to exhibit superb antiproliferative effects on cervical cancer cell lines (IC_50_ < 10 µg/mL). We must balance the promise of innovative research with a clear understanding of the challenges that need to be overcome for these therapies to be safe and effective in mainstream medical practice. Addressing these challenges and fostering continued research can bring us closer to realizing the full potential of EOs in cervical cancer care.

## Figures and Tables

**Table 1:** Common side effects of conventional treatment for cervical cancer.

Type of Treatment	Side Effects	References
Surgery	Lymphoedema, sexual and vaginal dysfunction	[24]
Chemotherapy	Vomiting, diarrhea	[25]
Immunotherapy	Hypothyroidism, liver toxicity	[26]
Radiotherapy	Rectal bleeding, constipation, hematuria, dysuria	[28]

**Table 3:** Summary of essential oil in treating cervical cancer in clinical trials.

Essential Oils	Study Design	Important Findings	References
Foot reflexology and aromatherapy	Randomized controlled trial of 41 locally advanced cervical cancer patients who received intracavitary brachytherapy	Average pain scores were lower for the intervention group. Average anxiety scores were lower for the intervention group.	[122]
Ginger aromatherapy	Pre-test post-test control group design of 60 post-cervical cancer chemotherapy patients	Improved nausea and vomiting frequency in the intervention group.	[123]
*Myrtus communis* L.-based vaginal suppository	Randomized double-blind placebo trial of 60 patients with cervicovaginal HPV infection	Increased HPV test negative results in the intervention group. Reduced cervical lesion size in the intervention group.	[124]
Antiviral AV2^®^ (mixture of eugenol, carvone, nerolidol, and geraniol in olive oil)	Randomized placebo-controlled clinical trial of 327 visual inspection of cervix with acetic acid-positive patients	No differences in regression of lesion and HPV clearance rate between intervention and control groups.	[125]

**Table 5:** Summary of essential oil in treating cervical cancer in clinical trials.

Conventional Drug	Essential Oils	Study Model	Important Findings	References
Bleomycin	Cinnamon oil nanoemulsion	HeLa cells	IC_50_ 10 μM Bleomycin IC_50_ 0.2 μM EO + Bleomycin Increased apoptotic effect compared to bleomycin alone.	[141]
Paclitaxel	Peppermint oil microemulsion	HeLa cells	Stable under centrifugal and freeze thaw stress. ≈90% of paclitaxel released in the first 48 h. Showed 70% and 90% viability reduction in HeLa cells after 24 and 48 h of exposure (greater than paclitaxel and EO alone).	[142]
Ifosfamide	Clove EO nanoemulsion	HeLa cells	IC_50_ 210 μM EO IC_50_ 140 μM EO + Ifosfamide	[143]
	Lemon EO nanoemulsion	HeLa cells	IC_50_ 7690 μM Ifosfamide IC_50_ 219 μM EO IC_50_ 165 μM EO + Ifosfamide	[144]
	Salvia EO nanoemulsion	HeLa cells	IC_50_ 7690 μM Ifosfamide IC_50_ 250 μM EO IC_50_ 141 μM EO + Ifosfamide	[144]
Mitomycin C	Chamomile EO nanoemulsion	HeLa cells	IC_50_ 29.8 μM Mitomycin C IC_50_ 1.4 μM EO IC_50_ 0.7 μM EO + Mitomycin C	[145]
	Frankincense EO nanoemulsion	HeLa cells	IC_50_ 10.59 μg/mL Mitomycin C IC_50_ 0.24 μg/mL EO + Mitomycin C	[146]
	Garlic EO nanoemulsion	HeLa cells	IC_50_ 29.8 μM Mitomycin C IC_50_ 1.8 μM EO IC_50_ 1.49 μM EO + Mitomycin C	[145]
	Ginger EO nanoemulsion	HeLa cells	IC_50_ 10.59 μg/mL Mitomycin C IC_50_ 0.36 μg/mL EO + Mitomycin C	[146]
Doxorubicin	Carvacrol-loaded chitosan NP	HeLa cells	IC_50_ 6.30 μg/mL Doxorubicin IC_50_ 2.98 μg/mL Carvacrol NP IC_50_ 5.66 μg/mL Carvacrol NP + Doxorubicin	[147]

EO, essential oil; IC_50_, half maximal inhibitory concentration; NP, nanoparticles.

## Data Availability

Not applicable.
